# Disruptive Effect of Organotin on Thyroid Gland Function Might Contribute to Hypothyroidism

**DOI:** 10.1155/2019/7396716

**Published:** 2019-04-17

**Authors:** Miriane de Oliveira, Bruna Moretto Rodrigues, Regiane Marques Castro Olimpio, Jones Bernardes Graceli, Bianca Mariani Gonçalves, Sarah Maria Barneze Costa, Tabata Marinda da Silva, Maria Teresa De Sibio, Fernanda Cristina Fontes Moretto, Lucas Solla Mathias, Dariane Beatriz Marino Cardoso, Helena Paim Tilli, Leandro Ceotto Freitas-Lima, Celia Regina Nogueira

**Affiliations:** ^1^Department of Internal Clinic, São Paulo State University (UNESP), Medical School, Botucatu, SP, Brazil; ^2^Department of Morphology, Federal University of Espírito Santo, Brazil

## Abstract

A considerable increase in endocrine abnormalities has been reported over the last few decades worldwide. A growing exposure to endocrine-disrupting chemicals (EDCs) can be one of the causes of endocrine disorders in populations, and these disorders are not only restricted to the metabolic hormone system but can also cause abnormal functions. Thyroid hormone (TH) disruption is defined as an abnormal change in TH production, transport, function, or metabolism, which results in some degree of impairment in body homeostasis. Many EDCs, including organotin compounds (OTCs), are environmental contaminants that are commonly found in antifouling paints used on ships and in several other industrial procedures. OTCs are obesogenic and can disrupt TH metabolism; however, abnormalities in thyroid function resulting from OTC exposure are less well understood. OTCs, one of the most prevalent EDCs that are encountered on a daily basis, modulate the thyroid axis. In most toxicology studies, it has been reported that OTCs might contribute to hypothyroidism.

## 1. Introduction

It is known that the human physiology and homeostasis depend on the precise functional harmony of different organs, as well as the proper functioning of the systems in the body. Together, the nervous and endocrine systems modulate and integrate the activities of almost all other body structures [1, 2]. The endocrine system consists of several glands, each of which is capable of detecting minimal hormonal variations in the bloodstream and thus regulating hormone synthesis/release; when its functioning is altered, homeostasis is immediately affected [2, 3].

A considerable increase in endocrine abnormalities has been reported over the last few decades, and a growing environmental exposure to endocrine-disrupting chemicals (EDCs) can be one of the causes of endocrine disorders in different populations worldwide [4]. According to the Environmental Protection Agency (EPA) of the USA and the World Health Organization (WHO), an EDC is defined as an exogenous mixture of substances that alters the function(s) of the endocrine system and consequently causes adverse health effects in an intact organism, its progeny, or in its populations [5, 6]. Most EDCs cause disruption through abnormal nuclear receptors signaling, which affects estrogen (ER*α* and ER*β*), progesterone (PR), glucocorticoid (GR), thyroid hormones (TR*α* and TR*β*), type retinoid X receptors (RXR*α*, *β*, and *γ*), and peroxisome proliferator-activated receptors (PPAR*α*, *β*/*δ*, and *γ*) [7, 8].

There are several chemicals, such as EDCs, that have the capacity to affect the endocrine system, including organotin compounds (OTCs). OTCs are synthetic chemical tetravalent derivatives of tin (IV) with a general formula *R*(4 − *n*) *S*_*n*_*X*_*n*_, where *R* represents an organic substituent and *X* a halide, anion, or an organic group linked covalently through a heteroatom (O, N, S, Cl, etc.) [9]. OTCs—a class of widespread, persistent EDCs with potent endocrine-disrupting properties in both invertebrates and vertebrates—have many industrial applications, which include their use as PVC catalysts and several biocide applications (agricultural fungicides, wood preservatives, and antifouling paints for marine vessels) [10, 11].

Many OTCs, including tributyltin (TBT), are related to reproductive toxicity in several species [12], causing imposex in mollusks (a syndrome where female gastropods develop male sex organs), fish alteration of their sex ratio in favor of males [13], and abnormal amphibian metamorphosis [14]. In addition, other abnormalities have also been reported in vertebrates species, for example, zebrafish (*Danio rerio*), when exposed to different doses of tributyltin chloride (TBTCl) from the first day of incubation of the eggs to PND 70, exhibited effects such as reduced or completely lost sperm motility, absence of flagella, and the presence of only abnormal spermatozoa in the semen [14]. Severe interstitial fibrosis was also observed in the interlobular septum of the testis when rockfish (*Sebastiscus marmoratus*) were exposed to 10 ng/L TBTCl; this resulted in testicular vacuolization at 48 days of exposure [15]. Thus, OTCs, as well as TBT, accumulate along food chains, leading to other abnormalities, which can affect humans [16].

Additionally, OTCs, as TBT, are considered obesogenic chemicals, which cause hyperplasia and hypertrophy of adipocytes and thus promote mammalian obesity via peroxisome proliferator-activated receptor gamma (PPARγ) signaling [17, 18]. However, adipogenesis and obesity are also observed in fish models. Den Broeder et al. [19] reported that exposing zebrafish embryos to a nontoxic concentration of tributyltin (TBT, 1 nM) from 0-6 days of postfertilization (dpf) and rearing the larvae to 15 dpf increased adipocyte differentiation and the expression of adipogenic genes such as *pparda*, *lxr*, and *lepa*.

Several studies of endocrine disruption have been carried out in the field, with a focus on estrogenicity and obesity, but the thyroid gland, thyroid hormone (TH) synthesis, and its signaling are now also recognized as important targets of OTCs' actions [20, 21].

The physiology of the thyroid is fundamental to the organism; in that, it is an endocrine gland that carries out its functions from the fetal life; its main function is to synthesize the TH, thyroxine (T4), and triiodothyronine (T3), which control the rate of metabolism of the body, thereby maintaining normal growth and development, as well as keeping body temperature and energy levels within their normal rates [22, 23].

Studies have reported that OTCs can disrupt the vertebrate hypothalamus-pituitary-thyroid (HPT) axis thyroid gland functions [4, 20, 24]. Environmental factors such as TBT, which exert complex toxicologic action on the HPT axis, can damage the thyroid gland and alter levels of TH, which might manifest as hypothyroidism [21].

## 2. Disruptive Effect of OTCs in the Thyroid Gland

The EDCs, including TBT, are one of the most important and toxic OTCs used to paint ships, as it reduces encrustation by algae, mussels, and other marine invertebrates; it is also included as a biocide in many disinfectants and insecticides [25].

OTC exposure has also been associated with various metabolic dysfunctions such as increase in body weight, vascular and neuropathological changes, and early puberty [26–28]. In particular, the TBTs, which are characterized as obesogens, cause metabolic abnormalities resulting in altered weight and obesity [29–31]. Grün et al. [17] showed that TBT is a potent inducer of adipogenesis, using both *in vitro* and *in vivo* mammalian models. TBT can interfere with the thyroid function, acting as a TH disruptor. Mammalian studies evaluating the effects of EDCs on the function of the HPT axis are limited. However, Andrade et al. [32] showed that in the female rat, TBT exposure caused critical abnormalities in the HPT axis, including decreased TRH mRNA expression and serum T4 levels; nevertheless, TSH level increased and did not alter T3. In addition to affecting thyroid morphology, EDCs were implicated in demonstrating decreased colloid and the thyroid follicle area with vacuolated follicular cells, which characterize follicular hypertrophy and hyperplasia. Also, TBT exposure might interfere with the peripheral TH metabolism; this is supported by the observed increase in type 1 deiodinase (D1) mRNA levels in the hypothalamus, which corroborates with studies carried out on zebrafish embryos using different chemical compounds that affected the thyroid, resulting in morphological and gene transcript expression changes [33].

Thyroid dysfunction is highly prevalent in the general population [34]. Data from a study of the American National Health and Nutrition Examination Survey (NHANES III) with 17,353 people aged ≥ 12 years suggest that hypothyroidism and hyperthyroidism are present in 4.6% and 1.3%, respectively, of the US population [35]. The correlation of TBT with estrogenicity and obesity is well defined in literature [20, 21], but TBT effects on the thyroid glands and their hormones are only partially understood.

In humans, TH disorders can modify cellular metabolism and can cause neurological impairment during fetal development [36, 37]. Several studies have reported the disruptive effect of OTCs in TH synthesis, TH metabolism, and TH serum transport in mammalian and nonmammalian species, mainly by TBT action which results in changes in the thyroid gland morphophysiological status [12, 17, 38, 39].

Rodrigues-Pereira et al. [40] verified TBT action on male Wistar rats that were treated orally with 500 ng/kg or 1000 ng/kg of TBT per day for 15 days. The authors found that animals treated with a larger dose of TBT showed apparent reduction in the follicle, colloid, and epithelium areas of the thyroid with a higher deposition of collagen. These structures may contribute to a loss of thyroid parenchyma organization [40]. In another recent study, male rats received a dose of 5 mg/kg TBT (5 × 10^6^ ng/kg) orally for 30 days. After this exposure, histopathological and ultrastructural changes were detected in the thyroid gland follicles. These changes included swollen and vacuolated appearance of the follicular cells, epithelial stratification, dilated rough endoplasmic reticulum filled with flocculent material, and an increased number of lysosomes. In addition, variation in the shape and size of the nuclei was also detected [4]. Andrade et al. [32] treated female Wistar rats for 40 days with 2 × 10^2^ ng/kg or 10^3^ ng/kg TBT dose, and their results showed abnormal thyroid parenchyma follicles of different sizes; several follicles with hypertrophied and hyperplastic epithelium were found.

Studies on nonmammalian vertebrates have also shown altered thyroid histology due to exposure to TBT or TBTCl; in a report by Zhang et al. [41], they were able to demonstrate decreases in follicle number and slight colloid reduction in the thyroid glands of fish (*Sebastiscus marmoratus)* at a TBT dose of 10 ng/L; however, at a dose of 100 ng/L, severe colloid depletion was observed.

Shi et al. [42] showed histological changes in the thyroid gland of an amphibian (*Xenopus laevis)* that was exposed to 200 ng/L TBTCl for 19 days. These included decreases in follicle number, the induction of hyperplasia and malformations, and colloid reduction.

These studies provide evidence for OTCs' detrimental effects on the thyroid gland through, in particular, the follicle cells, which are important for the adequate secretion of TH and may thereby cause homeostatic imbalance in important metabolic mechanisms.

## 3. OTCs Effects in TH Level

Maintenance of homeostasis requires the coordination of HPT, the TRH (which is essential in the metabolic homeostasis), and T3 (which integrates HTP signaling through a coordinated repression of TRH and gene expressions at the hypothalamic level) [8, 43, 44]. The evaluation of thyroid gland function is based on the assessment of serum TH and TSH levels [45]. Serum levels that are considered normal in the evaluation of thyroid function vary slightly according to the countries where analyses are performed.

Decherf et al. [8] verified the TRH gene promoter sequences in newborns upon exposure of TBT in pregnant rats and observed that TBT modifies the transcription of this promoter, affecting nuclear receptor signaling which modulates hypothalamic set points responsible for controlling metabolic responses. Shi et al. [42] showed in the amphibian *Xenopus laevis* that TBTCl significantly inhibited the metamorphosis and growth of tadpoles, confirming its disruptor effect on T3, an essential compound of this process in amphibians.

Sharan et al. [21] showed hypothyroid effect of TBTCl action on Swiss albino male mice, by T3, T4, TR*β*, and thyroid peroxidase (TPO) levels decrease, TSH increase, and transcriptional activity reduction of several thyroid differentiation genes. Moreover, they demonstrated that TBTCl might inhibit TR expression in human HepG2 cells, both by direct action and by corepressor/coactivator inhibition/increase, such as nuclear receptor corepressor (NCoR) and steroid receptor coactivator (SRC-1), respectively. In contrast, Rodrigues-Pereira et al. [40] verified TBT action on male Wistar rats and found that T4 levels were increased; this finding may be associated with type 2 deiodinase (D2) activity inhibition by TBT. The deiodinases, D1, D2, and D3, are responsible for the deiodination of T3 and T4 [46, 47]. The intracellular and plasma levels of TH are regulated by deiodinases, so a repression of D2 activity may lead to increased plasma T4 levels [40].

Male and female Wistar rats were fed bis(tri-n-butyltin) oxide (TBTO) at doses of 80 or 320 mg/kg for 4 weeks. Upon exposure to the lower dose, an increase in weight and adipose tissue and decreased T4 and TSH levels, in both male and female rats, were observed [39].

Wester et al. [39], using male and female Wistar rats that were exposed to dietary TBTO (0.5, 5, or 50 mg/kg diet) for 2 years, showed that there was a reduction in the level of free T4 in animals of both sexes that received high doses (5 or 50 mg/kg) of TBTO. In addition, the male rats that received the 50 mg/kg dose had lost weight, whereas the female rats that received the same dose maintained their weight. In agreement, other experimental studies with pregnant rats showed that when the animals were exposed to 0.25, 2.5, 10, or 20 mg/kg of TBT for 20 days during their gestational period, lower plasma levels of T3 and T4 were observed in only the 20 mg/kg dose [38].

Andrade et al. [32] treated female Wistar rats for 40 days with 2 × 10^2^ ng/kg or 10^3^ ng/kg TBT dose and showed an increase in D1 mRNA levels in the hypothalamus and thyroid by 10^3^ ng/kg TBT dose use, but a decrease in total T4 serum and T3 serum levels was not altered. TSH serum levels were increased by the 2 × 10^2^ ng/kg TBT dose use. The fish species, *Carassius auratus*, were exposed to 2.44 and 24.4 ng/L of TBT over 54 days. It was observed that at a lower level of TBT, the TH levels, as well as weight, were elevated; at a higher dose, weight and T4 levels were maintained and only T3 levels were reduced [12]. Other authors who worked with different fish species, besides demonstrating decreased T3 and T4 levels after TBT exposure in *Sebastiscus marmoratus*, also showed lower mRNA expression of TR*α* in the testes, which might affect the normal functioning of TH in spermatogenesis [41].

In female humans, one recent Danish study by Li et al. [48] provided some evidences about the associations between placental levels of various EDCs, including OTCs and TH. Thyroid function disorder leads to an impact on fetal and neonatal development, and thyroid function of fetuses can be affected in the uterus by EDCs. None of the OTCs investigated in the study had a significant association with T4 or T3 levels, whereas TBT concentration was inversely significantly associated with that of reverse T3 (rT3).

**Figure 1:**
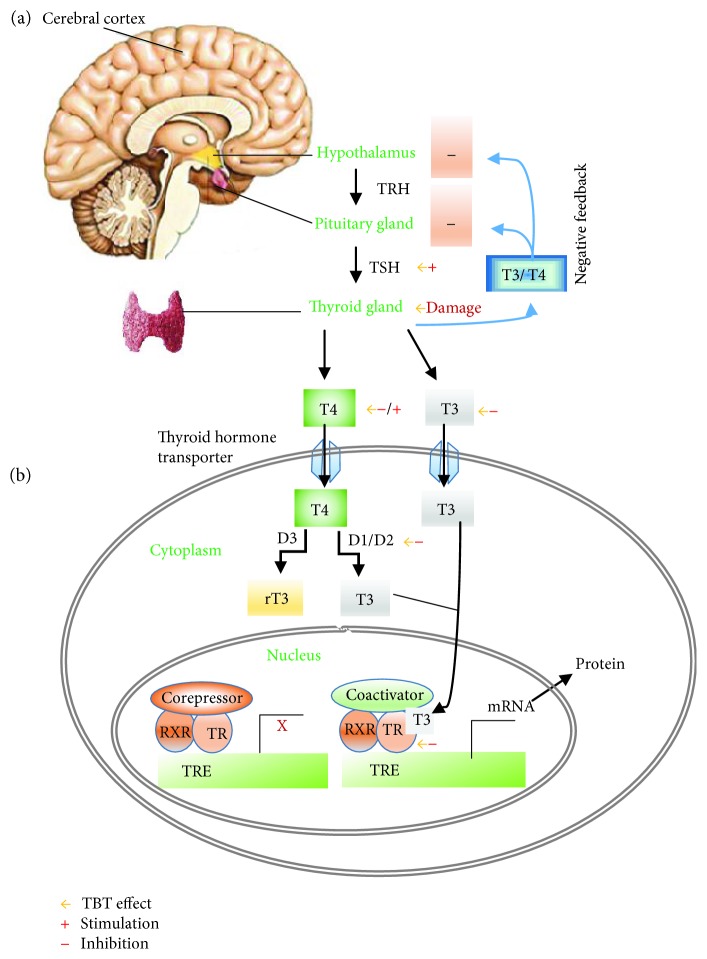
Production and action of thyroid hormones (TH) and a tentative representation of TBT disruption. The key components required for thyroid hormone action and possible TBT disruption are shown. (a) TH (T4 and T3) are produced by the thyroid gland and are regulated by thyroid-stimulating hormones (TSHs) produced by the hypophysis, which are stimulated by the thyrotropin-releasing hormones (TRHs). Once released, T4 and T3 exert a negative feedback mechanism on the production of TRH and TSH. TBT's disruptive effect on the hypothalamic-pituitary-thyroid (HPT) axis with stimulation of TSH or gland inhibition function and (b) the effects of T4 in vivo are mediated via T3; T4 is converted to T3 in target tissues by deiodinases 1 and 2 (D1 and D2). Deiodinase 3 (D3) converts T3 to the inactive T3 (rT3). T3 binding to the TR heterodimerizes with type retinoid X receptors (RXR) and binds to a TH response element (TRE), disrupting the corepressor binding and promoting the coactivator binding, which then leads to recruitment of polymerase III and the onset of gene transcription (mRNA). TBT's disruptive effect on thyroid in metabolic effect of T3 (inhibition) and T4 (diminished or incresead, if D2 is inhibited by TBT), with inhibition of deiodinases and TR or nonspecific linkage to TR.

These studies provide evidence for OTCs' disruptive effects on thyroid metabolism through the hypothalamic axis, TR, gland tissue development (as previously showed), and hormone production, such as that described and demonstrated in Table 1 and Figure 1, respectively; these TBT disruptive effects disturb thyroid physiological status.

## 4. Strategies to Inhibit the OTC-Disrupting Effects

The need to understand the potential effects of OTCs has led to a demand for *in vitro* and *in vivo* assay methods to identify the biological effects of a wide variety of natural and synthetic substances present in the environment; these compounds are defined by their activity and are thereby difficult to measure [49, 50].

A recent study demonstrated the development of a system to detect detrimental compounds at environmentally relevant levels by using a native estrogen receptor construct that is expressed on the surface of *Escherichia coli*. This approach should be broadly applicable for the detection of chemically diverse classes of compounds, including OTCs, that bind to a single receptor [51].

Liu et al. [52] demonstrated that TBT promoted both oxidative and DNA damages in mammalian cells by inducing oxidative damage to mouse cells both *in vivo* and in *vitro*. In a recent study, Badr El Dine et al. [4] investigated the toxic effects of TBT and found that it induced oxidative stress in rats as well as histopathological and ultrastructural changes in the thyroid. However, they found that a green tea extract, together with TBT, was useful in combating the tissue injury that resulted from TBT toxicity in the thyroid gland. It is known that green tea has antioxidant properties, owing to its ability to scavenge reactive oxygen species, which may have contributed to preserving thyroid function [53].

A literature review of potential biomarkers by Spaan et al. [33] analyzed the effects of 25 thyroid hormone disrupters which were observed on the zebrafish embryos, selected via the works of Weiss et al. [54] and Zhang et al. [55]. The effects of these compounds were compared across the studies, being limited to embryo exposures before 7 days of development. The most observed changes were morphological and related to the expression of the gene transcript; but no specific biomarker could be identified. More studies are needed to gain a better understanding of the mechanisms of HT disruption early in zebrafish development and its subsequent translation to humans in order to develop more convenient strategies to reduce the potential for the biological damage caused by chemical pollutants.

## 5. Conclusions

Although many studies have been carried out to detect EDCs in the environment, it is necessary to determine the levels that cause the reported effects. Further development in the identification and quantification of these substances is necessary, as they cause changes to the endocrine system at very low levels. It is important to study the disruptive effects of OTCs, especially TBTs, as they affect the organism as a whole. Even when affecting a single organ or the products of that organ, the changes can be reflected throughout the system.

TBT exposure affects the thyroid gland, causing damage, increased TSH, and reduction in the production of the thyroid hormones, T3 and T4. TBT and thyroid-related studies are still scarce in literature. However, they demonstrated the mechanisms involved in disruptive action effects of different levels of the HPT axis. The disruption affects TRH, TSH, TH (T3 and T4), deiodinase, and TRs, including the synthesis, metabolism, and the biological effects of THs in target tissues.

TBT is considered a toxic chemical when introduced in marine ecosystems; human exposure to this compound through dietary consumption could lead to a reduction in hormone levels, which could manifest as hypothyroidism, even though there are no reports of literature that strengthen this speculation.

In summary, this mini review clearly shows that OTCs, one of most prevalent EDCs that are encountered daily, modulate the thyroid axis, impairing its metabolism, and this confirmed reports from most studies that this chemical probably caused hypothyroidism.

## Figures and Tables

**Table 1:** TBT effects in the HPT axis and body weight in different species.

Species	Organotin dose/type	Time exposure	Hypothalamus		Pituitary		Thyroid					Reference
			Structural modification	TRH	Structural modification	TSH	Structural modification	T3	T4	Colloid	Follicles	
Male albino rats	5 × 10^6^ ng TBT/kg	30 days	nr	nr	nr	↑	Yes	↓	↓	↓	↓	[4]
Male Wistar rats	5 × 10^2^ ng TBT/kg	15 days	nr	nr	nr	nr	Yes	↔	↑	↔	↔	[40]
Male Wistar rats	10^3^ ng TBT/kg	15 days	nr	nr	nr	nr	Yes	↔	↑	↓	↓	[40]
Male Wistar rats	5 × 10^7^ ng TBTO/kg	2 years	Yes	nr	Yes	↔	Yes	nr	↓	nr	↓	[39]
Female Wistar rats	5 × 10^7^ ng TBTO/kg	2 years	Yes	nr	Yes	↔	Yes	nr	↓	nr	↓	[39]
Female Wistar rats	2 × 10^2^ ng TBT/kg	40 days	nr	↓	nr	↑	Yes	↔	↔	↔	↔	[32]
Female Wistar rats	10^3^ ng TBT/kg	40 days	nr	↓	nr	↔	Yes	↔	↓	↓	↓	[32]
Male Swiss albino mice	5 × 10^2^ ng TBTCl/kg	45 days	nr	nr	nr	↑	No	↔	↔	↔	nr	[21]
Male Swiss albino mice	5 × 10^3^ ng TBTCl/kg	45 days	nr	nr	nr	↑	Yes	↓	↓	↓	nr	[21]
Male Swiss albino mice	5 × 10^4^ ng TBTCl/kg	45 days	nr	nr	nr	↑	Yes	↓	↓	↓	nr	[21]
Female Sprague-Dawley rats	2.5 × 10^5^ ng TBTCl/kg	20 days	nr	nr	nr	nr	nr	↔	↔	nr	nr	[38]
Female Sprague-Dawley rats	2.5 × 10^7^ ng TBTCl/kg	20 days	nr	nr	nr	nr	nr	↔	↔	nr	nr	[38]
Female Sprague-Dawley rats	10^7^ ng TBTCl/kg	20 days	nr	nr	nr	nr	nr	↓	↓	nr	nr	[38]
Female Sprague-Dawley rats	2 × 10^7^ ng TBTCl/kg	20 days	nr	nr	nr	nr	nr	↓	↓	nr	nr	[38]
Xenopus tropicalis	12.5 ng TBTCl/L	7 days	nr	nr	nr	nr	Yes	nr	nr	↔	↔	[42]
Xenopus tropicalis	12.5 ng TBTCl/L	19 days	nr	nr	nr	nr	Yes	nr	nr	↔	↔	[42]
Xenopus tropicalis	50 ng TBTCl/L	7 days	nr	nr	nr	nr	Yes	nr	nr	↔	↔	[42]
Xenopus tropicalis	50 ng TBTCl/L	19 days	nr	nr	nr	nr	Yes	nr	nr	↔	↔	[42]
Xenopus tropicalis	2 × 10^2^ ng TBTCl/L	7 days	nr	nr	nr	nr	Yes	nr	nr	↔	↔	[42]
Xenopus tropicalis	2 × 10^2^ ng TBTCl/L	19 days	nr	nr	nr	nr	Yes	nr	nr	↓	↑	[42]
Carassius auratus (goldfish)	2.44 ng TBT/L	54 days	nr	nr	nr	nr	nr	↑	↑	nr	nr	[12]
Carassius auratus (goldfish)	24.4 ng TBT/L	54 days	nr	nr	nr	nr	nr	↓	↔	nr	nr	[12]
Male S. marmoratus	1 ng TBT/L	50 days	nr	nr	nr	nr	No	↓	↔	↔	↔	[41]
Male S. marmoratus	10 ng TBT/L	50 days	nr	nr	nr	nr	Yes	↓	↓	↓	↓	[41]
Male S. marmoratus	10^2^ ng TBT/L	50 days	nr	nr	nr	nr	Yes	↓	↓	↓	↓	[41]

HPT: hypothalamus-pituitary-thyroid; nr: not reported; ↔: unchanged; ↑: increased; ↓: decreased; TBT: tributyltin; TBTO: bis(tri-n-butyltin) oxide; TBTCl: tributyltin chloride; TRH: thyrotropin-releasing hormone; TSH: thyroid-stimulating hormone; T3: triiodothyronine; T4: thyroxine.
